# The Influence of Covid-19 on Perceived Health Effects of Wetland Parks in China

**DOI:** 10.1007/s13157-021-01505-7

**Published:** 2021-10-25

**Authors:** Xuezhu Zhai, Eckart Lange

**Affiliations:** Department of Landscape Architecture, University of Sheffield, Western Bank, Sheffield, S10 2TN UK

**Keywords:** Health, COVID-19, Wetland park, Perception, Ecosystem services

## Abstract

Wetland parks are designed to support urban ecological protection, flood control and human well-being. Existing research mainly focuses on their influence on ecology and economy. However, their influence on human well-being and health is rarely studied. In China, during the peak of the COVID-19 pandemic (Peak), people were very concerned about health, while at the same time wetland parks which are generally considered beneficial to health were closed. Thus, this study explores the public’s perception of the health effects of visiting wetland parks and the impact of the pandemic on the perception. From March 5th to 8th, 2020, before the Peak in China was over, 1,400 respondents participated in a nationwide online survey. It was found that the perceived benefits from visiting wetland parks were higher in terms of mental health than in physical health. Also, the perceived health benefits of wetland parks after the Peak were slightly higher than before the pandemic. The results highlight that wildlife habitat services were considered to be the most important ecosystem services that promote the perceived health benefits. Interestingly, the perceived health benefits of wetland parks by health experts appear to be lower than in other groups, indicating that the health benefits of visiting wetland parks may be overestimated by lay-people or underestimated by health experts. The results provide empirical evidence for managing ecosystem services as delivered by these urban wetlands, in the context of COVID-19 or potential future pandemics, for promoting public health.

## Background

**Fig. 1:**
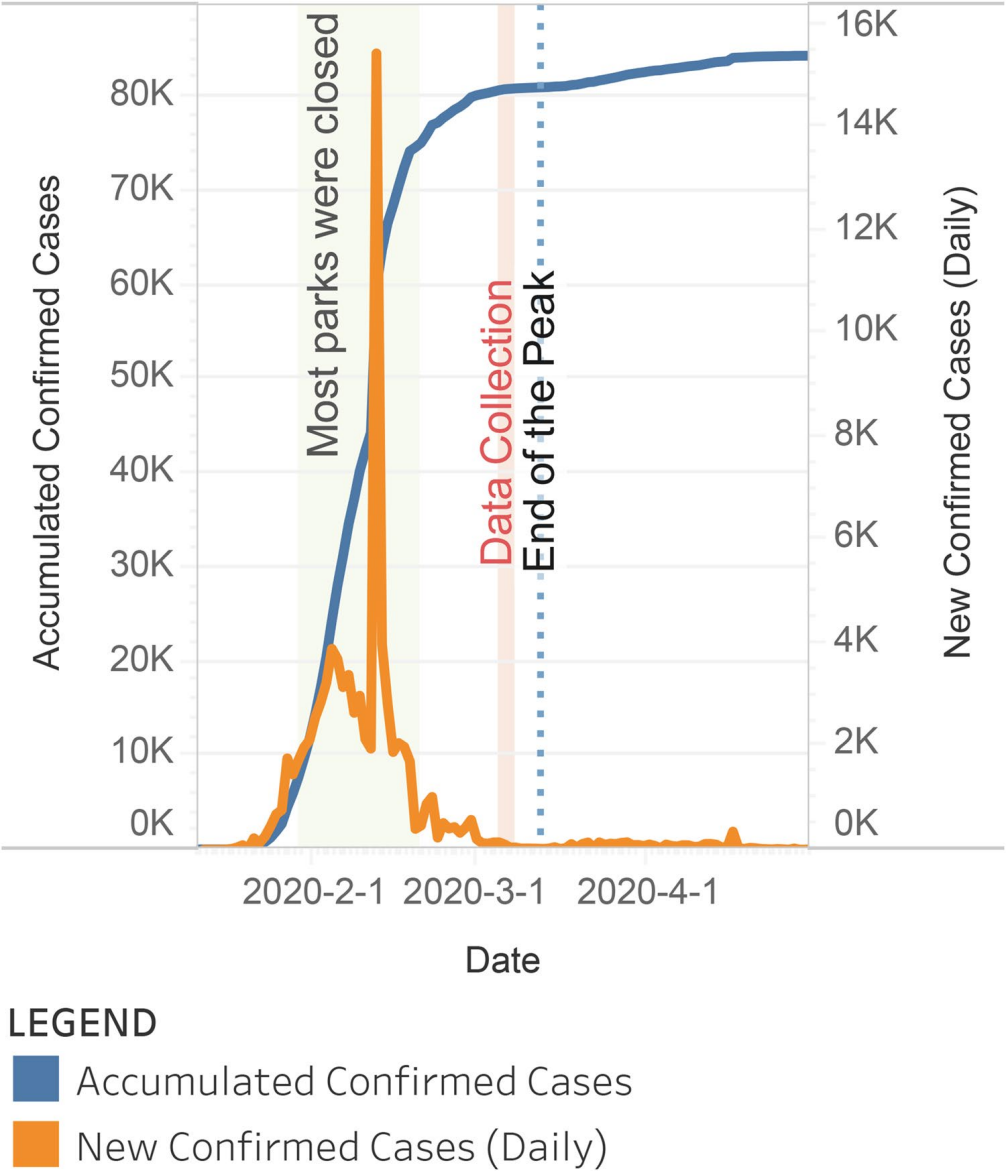
Timeline of the COVID-19 in China (Data source: DX Doctor COVID-19 Pandemic Real-time Report), milestones relevant to this study, and the data collection period

Urban dwellers in China experienced profound levels of anxiety and poor perceived health during the peak of the COVID-19 (Peak) (Ni et al. 2020), from January to March in 2020 (shown in Fig. 1). During the worst month of the Peak, many cities were locked down, and most parks were shut down. After February 17^th^, some employees started to return to work, but non-essential travel was not encouraged. After February 21^st^, when CHSLA (Chinese Society of Landscape Architecture (CHSLA) 2020) published a Group Standard for guiding operational management of urban parks during the pandemic, some parks began to reopen. Subject to compliance with the Standards, visitors were limited to 30–50% of the carrying capacity. Data for this study were collected from March 5^th^ to March 8^th^, when the curve of the pandemic dropped steeply and hit the bottom in terms of new confirmed cases. It was just a few days before the official announcement^1^ of the end of the Peak on March 12^nd^ (Zou 2020), when the case numbers were similar to the end of the Peak and most epidemic prevention measures were lifted.

### Wetland Parks, Health, and Human Well-being

#### Wetland Parks

In this study, according to the Classification Standard for Urban Green Spaces (Ministry of Housing and Urban–Rural Development of the People’s Republic of China (MOHURD) 2017), wetland parks (WPs) include not only ecological parks with "wetland parks" in the name but also public green spaces containing rivers, lakes and other wetlands.

#### Health Effects of Wetland Parks and Ecosystem Services

Many studies have shown that natural environments can be beneficial to physical and mental health. For example, exercising in natural environments brings higher levels of happiness than exercising in indoor and street environments (Bowler et al. 2010; Olafsdottir et al. 2017), 5]. Contact with nature could affect health in many ways, e.g. fresh air, physical exercise, social cohesion, and stress reduction (Hartig et al. 2014).

For most people living in cities, urban green spaces are the most (sometimes the only) accessible natural resource (Maller et al. 2010). Many scholars have evaluated the health effects of green spaces around the living environment, and found that (1) there is a positive or weak correlation between green space and obesity-related health (Lachowycz and Jones 2011); (2) the higher the ratio of green space in community, the lower the risks of mental health risks and cardiovascular disease (Richardson et al. 2013), and the higher the self-rated health status (Orban et al. 2017); (3) and the ratio of urban green space in a city is negatively correlated with the rate of local antidepressant prescriptions (Helbich et al. 2018). It also has been proved that urban green spaces can promote Chinese residents’ physical activity so as to improve public health (Wang et al. 2019).

Urban blue space and proximity to water also promotes human health (Crouse et al. 2018). Specific to the wetland ecosystems, they can promote human well-being and health by provision of safe drinking water, improving resilience to natural disasters, and providing medicines; but it may also harm health by spreading diseases and releasing pollutants (Horwitz and Finlayson 2011).Besides, experiencing the physical and mental health benefits of healthy wetlands can offset some of the stress and illness associated with disasters such as flooding, drought, and wildfires (Sutton-Grier and Sandifer 2019). These health benefits can be attributed to ecosystem services (ESs) including provisioning, regulating and cultural, helping with e.g. malignant neoplasms, mental and behavioural disorders, and cardiovascular disease (Oosterbroek et al. 2016). Despite these fragmentary evidences, the health effects of WPs—a particular type of urban wetlands—are poorly understood, in particular regarding how they are perceived when visited and experienced. As Scholte et al. (2016) suggest, understanding how people interact with ecosystems is important to foster public support for wetland restoration; learning how the public perceive the health benefits from wetland parks could help with fostering public support for urban wetland restoration.

### Perceived Health Effects

Urban environments and their perception significantly affect residents' self-evaluated health. Urban greening and infrastructure conditions are the main influencing factors (Wang et al. 2020). The expected benefits to human health, especially the expected improvement in psychological and social welfare, of visiting nature reserves are considered to be the main value of personal preference and choice of visiting nature reserves (Lemieux et al. 2012). Besides, people are increasingly aware of the positive relationship between visiting parks and nature reserves and related health benefits (Romagosa et al. 2015). Also, different people may have different perceptions of the health effects of the same environment, so it is of great significance to study the perception of the health effects of diverse populations.

### Aims

The main aim of the research is to (1) explore the public's perception of the health effect of WPs before, during, and after the peak of the COVID-19 pandemic (Peak), and (2) explore the impact of the epidemic and other factors on people's perception of health effect of WPs.

## Methods

### Data Collection: Online Questionnaire

The data for the study was collected nationwide in China through online questionnaires using the Tencent Questionnaire platform. The differences in pandemic risks across provinces were used to study the impact of the epidemic on perceived health effects. The questionnaire was distributed using snowball sampling on the social media WeChat, which has the largest number of users in China (with 1.21 billion monthly active users in 2020) as the "seed", from March 5th to 8th, 2020. We set the sample size to 1400 (one out of 100,000 of the total population of China), considering that when the sample size increases to 1000, the sharp increases in precision due to the growth of sample size becomes less pronounced (Bryman 2012). Also, after deducting non-wetland park users, the sample size could be large enough for a margin of error between 3 and 5 with a 95% confidence level. (Hazra 2017) Once the target number of total valid responses (1,400) was reached, data collection was stopped.^2^

After collecting demographic data, questions of "whether you would like to visit a park/WPs after the pandemic is over?"^3^ were asked separately; the survey would continue if the respondents indicated a willingness to visit WPs. At the beginning of the questionnaire and in the note of each question include "WPs", the definition of WPs was given with examples of well-known WPs: The "wetland parks" in this survey include both ecological theme parks with "wetland parks" in their names (such as Hangzhou Xixi National Wetland Park, Suzhou Tai Lake National Wetland Park, Wuhan East Lake National Wetland Park, Guangzhou Nansha Wetland Park, etc.), as well as including parks dominated by wetlands such as rivers and lakes with good ecological functions (such as Shenzhen Dasha River Park, Guangzhou Lu Lake Park, Chengdu Living Water Park, etc.). Participants who were unwilling to visit parks and WPs were asked to give reasons, and then skip to the end of the survey.

For respondents who would like to visit WPs, questions about the frequency of visit before and after the pandemic were asked: (1)" If wetland parks were not closed during the epidemic, and your community and nearby roads were not closed, would you visit wetland parks?"; (2) "After the outbreak, how often do you think you will visit wetland parks?"; (3) "After the epidemic, what do you think is the reason why your frequency of visiting wetland parks would be increased or decreased (Please skip this question if you would not change your frequency of visits)?" The survey continued only when a respondent had been to wetland parks in the year before the outbreak. Also, respondents were asked to name their favourite WP, and reasons why this WP was preferred was asked using a multiple-choice question with an "others" option for the participants to respond.

These were followed by a set of questions about willingness to visit WPs during the Peak: (1) “If the wetland park was not closed during the epidemic, and your community and nearby roads were not closed, would you visit wetland park? (a single choice question)”; (2) “Whether the wetland park you usually go to, or you last visited has been reopened? (a single choice question)”; (3) (multiple choice question with “other” option only for respondents who chose the option “it has been opened orderly and you have been to” in the last question) “After the orderly opening of wetland parks, even the procedures are complicated, and masks are needed, why do you still visit the wetland parks?”.

Other independent variables (influencing factors at four levels) and dependent variables (perceived health effects related to WPs) were collected, as described in the following sections. Respondents spent an average of 6.5 minutes filling out the questionnaire. Two rounds of pre-tests were conducted before March 5^th^ to ensure that respondents correctly understand the questionnaire.

### Dependent Variable: Perceived Health Effects Associated with Wetland Parks

This study uses perceived health benefits or risks as dependent variables to characterize the impact of wetland parks on health perceived by citizens. A seven-point Likert Scale was used to evaluate the perceived mental and physical health effects of visiting WPs before, during, and after the Peak. Respondents were asked "Before/During/After the Peak, what do you think will be the impact of visiting WPs on your physical/mental health?" respectively.

### Independent Variables: Factors of Perceived Health Effects

This study included four levels of variables, namely city, community, WPs, and individual levels.

#### City Level

During the Peak, the severity of the epidemic situation (i.e., the numbers of cumulative confirmed cases, newly confirmed cases and deaths) varied among provinces and cities in China, leading to different epidemic risks and emergency policies. These may affect the perceived health effects of wetland parks. The Response Level to Public Health Emergency (RLPHE) in a given region on a single day can reflect the risk level of an outbreak in that region on that day. The investigation period was at the end of the Peak, and some areas where the outbreak was not severe (i.e., there were not many confirmed cases and there had been no newly confirmed cases for a while) have lowered the RLPHE.

By asking about the main cities of residence at the peak of the epidemic, and according to the RLPHE of all provinces and cities across the country on March 6^th^ (midpoint in the sampling period), these cities were classified into three categories: first-level response, namely the highest risk; second-level response, high risk; third-level response, medium risk.

#### Community Level

During the Peak, many communities in cities with higher epidemic risk levels were locked down. Some communities were entirely locked down, and quarantine was required. Some communities were semi locked down, where residents could leave their homes and do activities in the communities, but could not go out of the community unless necessary. In low-risk cities, the communities were not closed. Information on the degree of community lockdown during the Peak was collected using a single-choice question.

#### WPs Level

##### Health Effects of Wetland Parks

Respondents were asked about the name of the wetland park they often visited or their favourite and why they like this WP. The wetland parks that the participants visited most or their favourites were coded according to the main wetland types they contain (e.g., lakes, rivers, coast, swamp), and the correlation analysis of preferred wetland types in the same corresponding level of regions with health effect perception (measured in "Dependent Variable: Perceived Health Effects Associated with Wetland Parks" section) was carried out to study the perceived health effects of preferred wetland types.

##### Health Effects of Ecosystem Services

This study explored the perceived ESs from wetland parks and the health effect of these perceived ESs, by asking participants to make multiple choices for perceived ESs first, and then ranking their choices according to importance to the improvement of their physical and mental health. ESs including habitat, water purification, air purification, noise reduction, flood regulation, recreation, aesthetics, education, and social relations were involved. These examined ESs were selected according to previous studies on perceived ESs in WPs (Zhai and Lange 2020). They belonged to the regulating, cultural and supporting category. The provisioning services were not examined in this study because WPs do not always deliver provisioning services (e.g., food, raw materials).

#### Individual Level

This part first collected the respondents' socio-demographic details (such as age, gender, highest education level, professional, occupation status, and city of residence) through five single-choice questions and two drop-down questions. Respondents' self-reported physical and mental health status before and during the Peak was then collected through four five-point Likert scale questions.

### Data Analysis

All statistical analyses were performed using SPSS Statistics 25. Descriptive statistics were used to analyze the respondents’ profiles. The open-ended questions were coded for descriptive statistics. Analysis of variance (ANOVA) and one-way T-test were used to examine whether various factors affect perceived health effects. Bivariate correlation analysis was used to study the correlation between self-reported health status and perceived health effects.

## Results

### Respondents’ Profiles

**Fig. 2:**
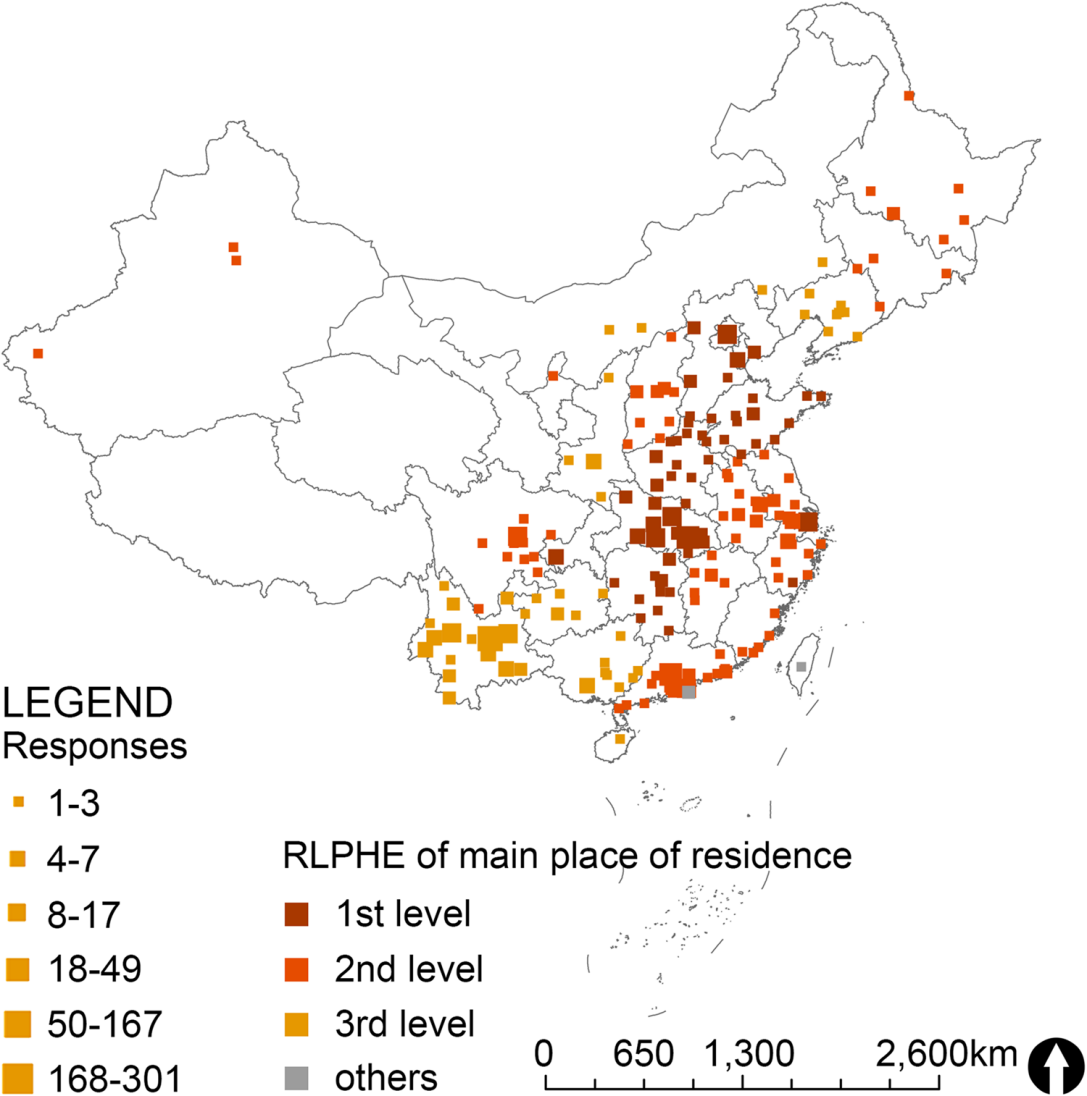
Geographical distribution of respondents

The majority of the respondents were young and middle-aged (65.43% of respondents were younger than 34 years old) and have a high level of education (graduate or higher) (Table 1). 57.9% of the respondents were females. 63.0% of the respondents were employed, 27.8% were students, and others were retired or unemployed. 43.2% were engaged in architecture and built environment, and 6.14% were health experts (i.e., medical and nursing or psychology professionals). Respondents came from 31 provinces including 161 cities, and were evenly distributed in cities with the three levels of RLPHE (Fig. 2). During the Peak, 75.3% of the respondents lived in semi-lockdown communities, 18.8% were quarantined at home, and 5.9% had free access to their homes and communities.

**Table 1:** Respondents’ profile

	N = 1400	(%)	2010 Census (%)
Age			
18–24	362	25.86	15.31
25–34	554	39.57	18.21
35–44	232	16.57	19.21
45–54	174	12.43	14.03
55–64	65	4.64	9.34
≥ 65	13	0.93	7.68
Gender			
Male	590	42.14	51.14
Female	810	57.86	48.86
Highest Education Level			
Junior high school and below	20	1.43	46.56
High school equivalent	80	5.71	22.43
Specialized college	118	8.43	11.65
Bachelor's	655	46.79	9.37
Master's and above	527	37.64	1.02
Occupation Status			
Student	389	27.79	-
Employee	882	63	-
No occupation	62	4.43	-
Retiree	67	4.79	-
Professional			
Architecture & built environment	605	43.21	
Art & design	129	9.21	
Hydrology	21	1.5	
Psychology	14	1	
Medicine, nursing	72	5.14	
Agriculture/forestry	56	4	
Environmental science	27	1.93	
Social science	73	5.21	
Economy & finance	60	4.29	
Others	343	24.5	
RLPHE of main place of residence			
1st level	473	33.79	34.32%
2nd level	392	28.00	50.38%
3rd level	530	37.86	15.31%
Community closure status			
Totally lockdown	263	18.79	-
Semi lockdown	1054	75.29	-
Not lockdown	83	5.93	-

2010 Census means the population census of the People’s Republic of China in 2010, to show the representativeness of the samples. Data source: National Bureau of Statistics of People’s Republic of China. Although it is known that the online population is usually different from the total population, which makes it difficult for the online questionnaire sample to represent the total population, we have compared the demographic information of the sample with the national census to understand the difference between the sample and the total population

### Willingness to Visit Wetland Parks

**Fig. 3:**
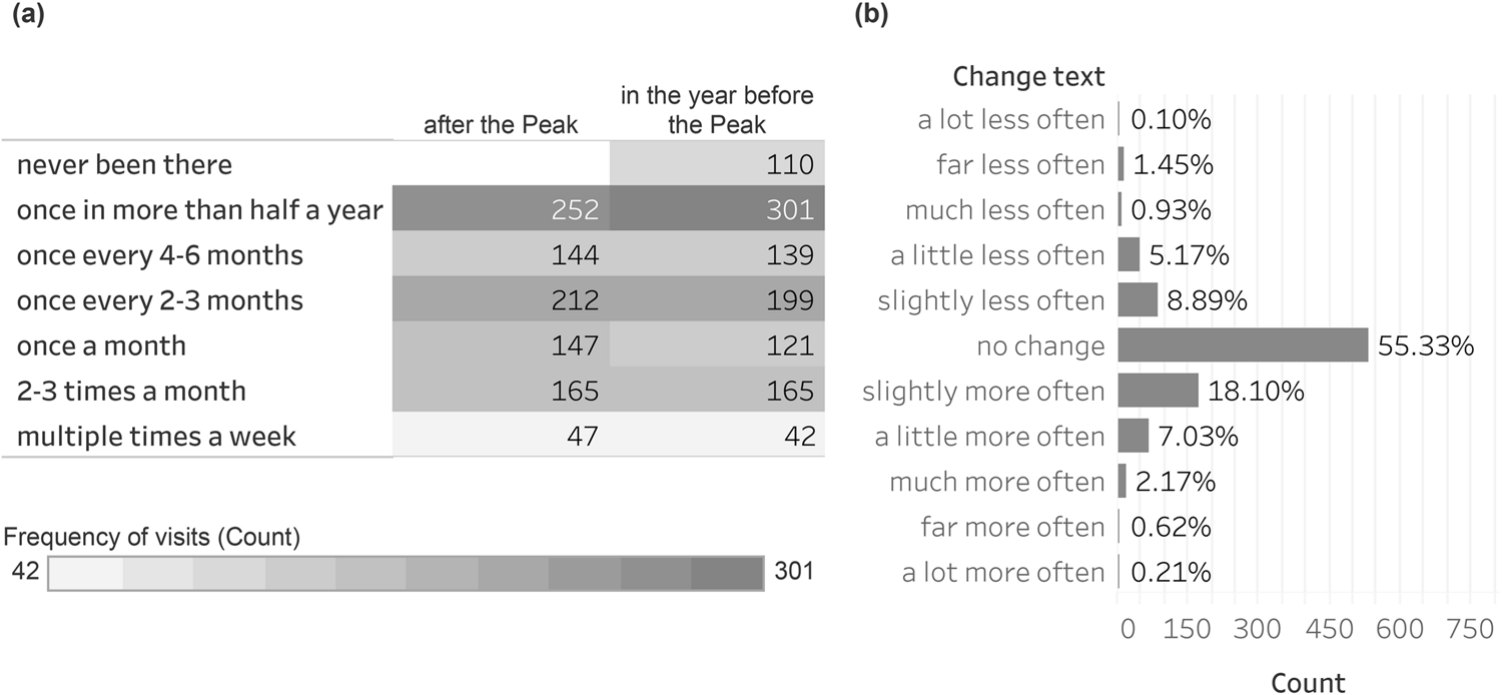
Frequency of WPs visits: in the year before the Peak, expected after the Peak, and the change

**Fig. 4:**
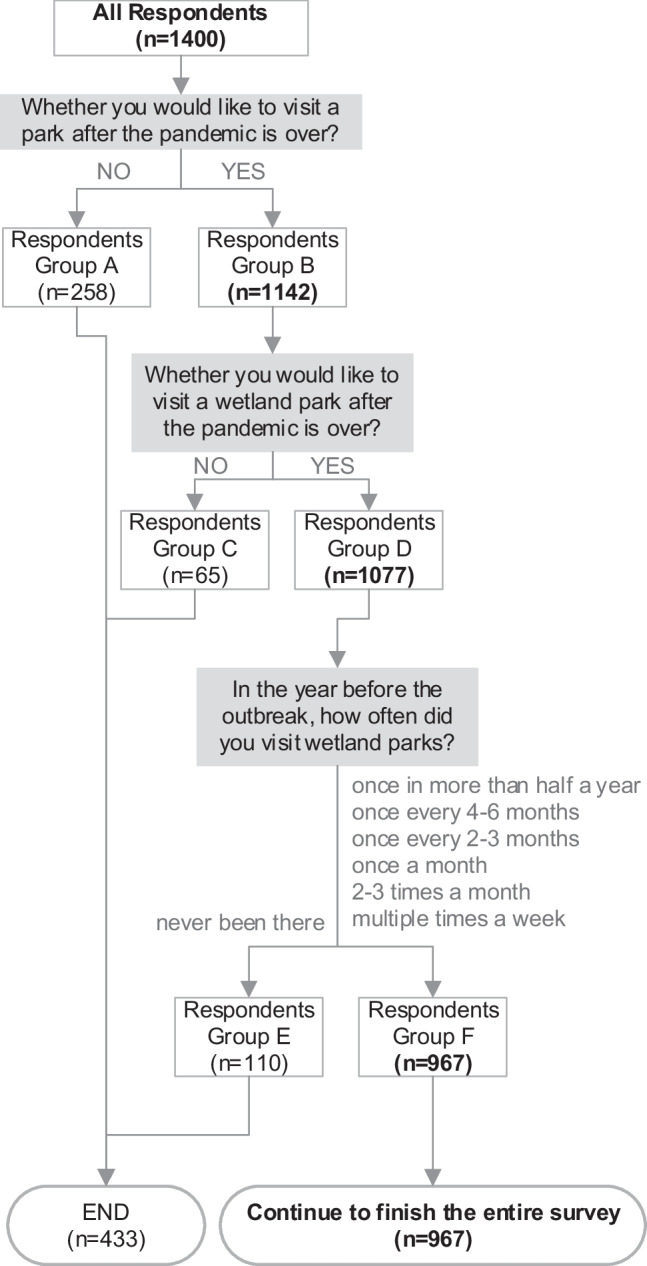
Responses filters

81.6% of the respondents were willing to visit parks after the Peak (N = 1142). 76.9% of those who wanted to visit parks also wished to visit WPs (N = 1077). The main reasons for not visiting WPs were poor accessibility (52.3%). After the Peak, 28.2% of the respondents would increase their visiting frequency, while 55.3% of respondents would keep their visiting frequency (Fig. 3). Among the 1077 respondents who wished to visit WPs, 110 respondents had not been to wetland parks in the year before the outbreak. Considering that those 110 respondents may not be familiar with WPs, they were excluded from the following sections of survey. Thus, there were 967 respondents in total who took part in the whole survey (Fig. 4).

**Fig. 5:**
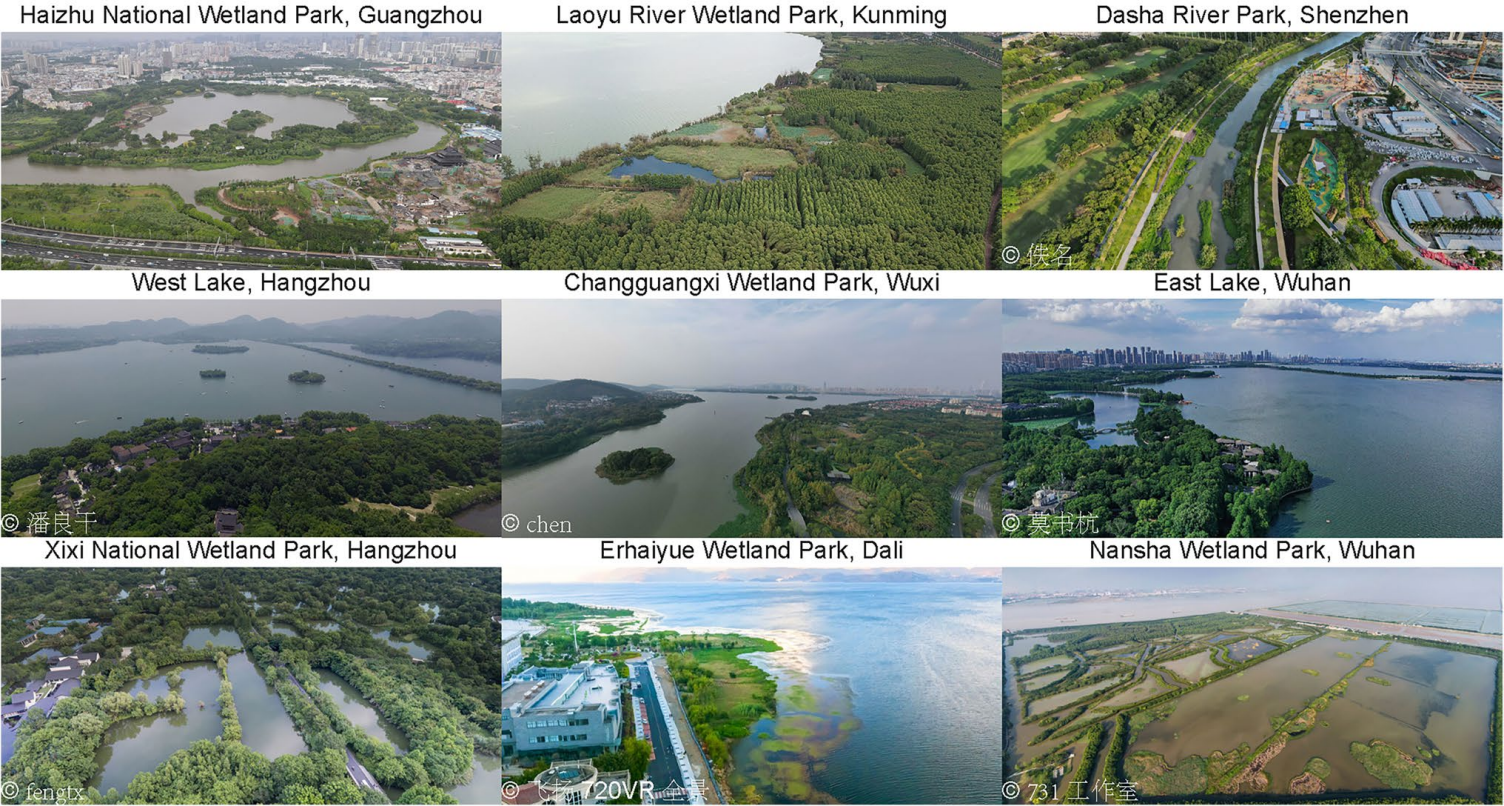
Wetland parks that respondents mentioned most frequently. (Pictures marked with copyright are source from: www.720yun.com)

A total of 109 respondents had visited WPs (e.g., Fig. 5) since they reopened after the peak of the epidemic within two weeks. Fresh air (57.8%), physical exercise (43.1%), and exposure to nature and wildlife habitats (42.2%) were the main motivations. ‘WPs are sparsely populated with low risk of infection’ (36.7%), ‘Basking in the sun and enjoy the breeze’ (35.8%) and ‘enjoy the beautiful scenery’ (29.4%) were important driving factors.

### Dependent Variables: Perceived Health Effects

**Fig. 6:**
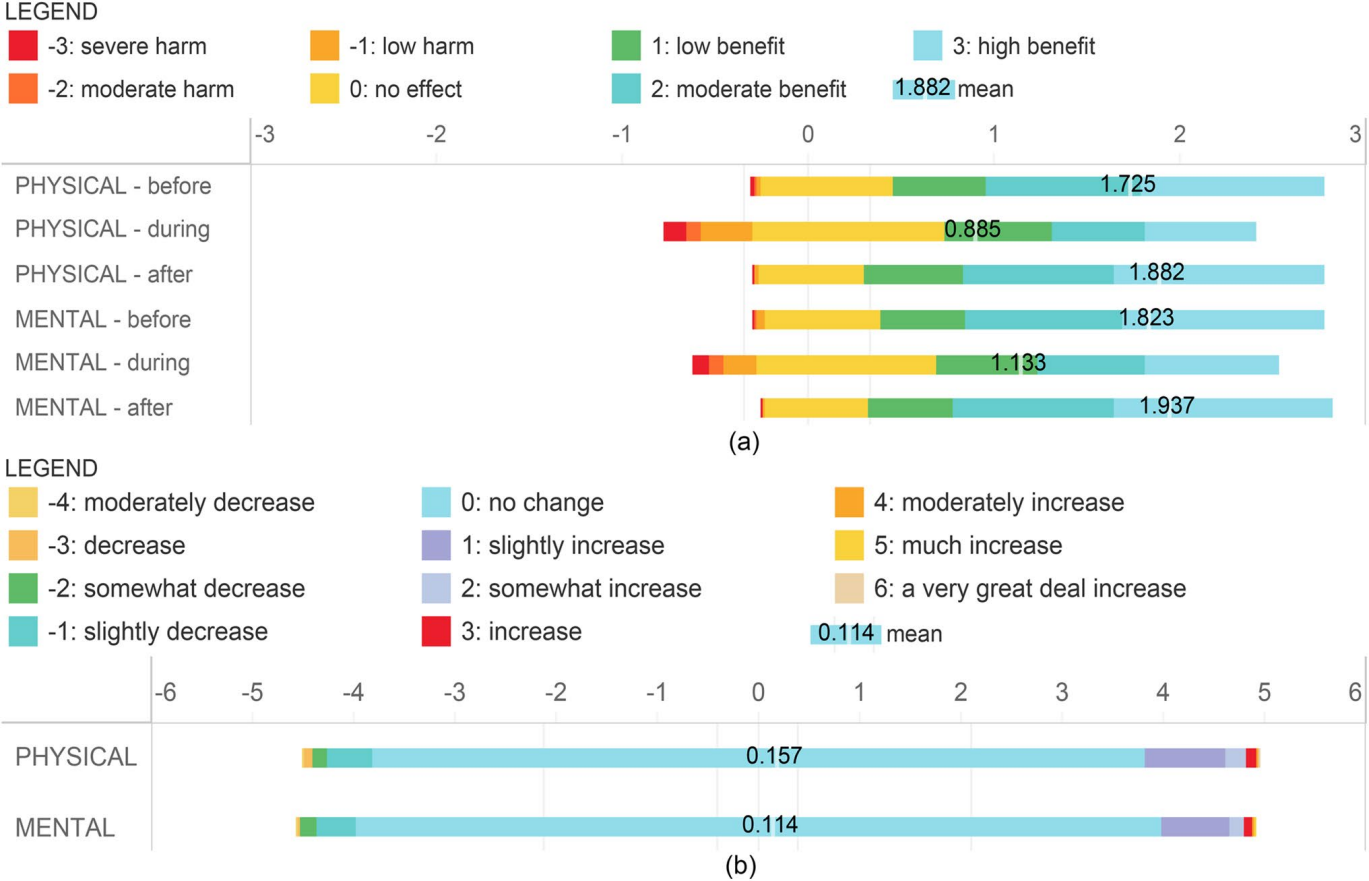
Perceived health benefit before, during and after the peak of the COVID-19 outbreak. Divergent Stacked Bar: The length of each colour represents the proportion of respondents who chose this attitude to the total number of respondents. The starting point of each bar graph is different, and the total length is 100%

The set of health-relevant scale items passed the reliability test (Cronbach’s alpha = 0.797) and the validity test (KMO measure of sampling was adequate (= 0.732), and Bartlett’s test of sphericity was significant (P = 0.000)). The results (Fig. 6) show that people perceive health benefits from WPs; even during the peak of the epidemic when the perceived benefits were the lowest, benefits still outweigh potential risks. The perceived benefits of visiting wetland parks on mental health were higher than that on physical health, especially during the peak of the epidemic. The perceived health benefits expected after the Peak were slightly higher than before the Peak: approximately 70% of the respondents perceived the same level of health effects from WPs before and after the Peak; about 20% of the respondents believed that health benefits have increased after the Peak; in contrast, about 10% of the respondents assumed that perceived health benefits decreased.

### Independent Variables

#### City Level

As shown in Table 2, the RLPHE of the city of residence had a significant impact on the perceived physical and mental health benefits during the Peak and on the perceived mental health benefits after the Peak (P < 0.05). Respondents in the second RLPHE regions perceived the highest health benefits, and those in the first RLPHE areas (the highest-risk area) perceived the lowest physical health benefits during the Peak and the lowest mental health benefits after the Peak. In contrast, respondents in third RLPHE regions (medium-risk areas) perceived the lowest mental health benefits during the Peak. On average, the perceived health benefits from WPs after the Peak were slightly higher than before the outbreak in all the three types of regions, but there was no significant difference in the change of perceived health benefits in these regions.

**Table 2:** Impact of the factors (city and community level) (*: P < 0.05, **: P < 0.01)

	N = 967	Before Peak		During Peak		After Peak		Difference:	
								After-before Peak	
		Physical	Mental	Physical	Mental	Physical	Mental	Physical	Mental
CITY LEVEL: RLPHE of main place of residence									
1st level	314	1.63 ± 1.28	1.71 ± 1.24	0.79 ± 1.56	1.09 ± 1.50	1.81 ± 1.18	1.87 ± 1.18	0.18 ± 1.13	0.16 ± 0.89
2nd level	260	1.82 ± 1.20	1.97 ± 1.13	1.12 ± 1.55	1.43 ± 1.43	2.01 ± 1.10	2.08 ± 1.04	0.19 ± 1.03	0.12 ± 0.87
3rd level	389	1.74 ± 1.25	1.82 ± 1.25	0.81 ± 1.58	0.97 ± 1.60	1.86 ± 1.67	1.90 ± 1.15	0.12 ± 0.96	0.08 ± 0.89
One-way ANOVA	F	1.73	3.145	3.864	7.167	2.413	3.059	0.406	0.7
	P	0.178	0.043	0.021*	0.001**	0.09	0.047*	0.666	0.497
COMMUNITY LEVEL: Community closure status									
Totally lockdown	178	1.58 ± 1.30	1.60 ± 1.31	0.83 ± 1.63	1.12 ± 1.49	1.67 ± 1.25	1.70 ± 1.24	0.09 ± 1.05	0.10 ± 0.95
Semi lockdown	732	1.75 ± 1.23	1.86 ± 1.19	0.88 ± 1.56	1.14 ± 1.54	1.91 ± 1.14	1.98 ± 1.11	0.16 ± 1.04	0.11 ± 0.86
Not lockdown	57	1.81 ± 1.25	2.00 ± 1.18	1.09 ± 1.57	1.09 ± 1.53	2.19 ± 0.99	2.19 ± 0.99	0.39 ± 0.92	0.19 ± 0.91
one-way ANOVA	F	1.442	3.994	0.601	0.035	5.202	5.917	1.774	0.27
	P	0.237	0.019*	0.548	0.966	0.006**	0.003**	0.17	0.764

#### Community Level

The lockdown level of the respondents’ community during the Peak had a significant impact on the perceived physical and mental health benefits after the peak of the epidemic (P < 0.01) (Table 2). Surprisingly, as the degree of community lockdown level increased, the expected perceived physical and mental health benefits after the peak of the epidemic decreased. Because community lockdown occurred after the outbreak, the differences in the perceived level of mental health benefits before the Peak was not considered to be caused by community lockdown.

#### Wetland Parks Level

##### ESs for Promoting Perceived Health Benefits

**Fig. 7:**
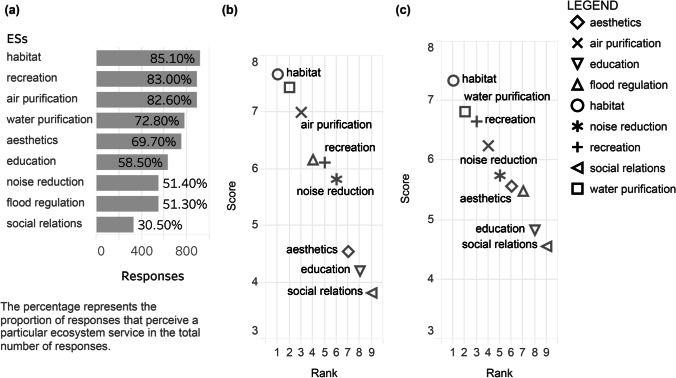
Perceived ESs in WPs and ranking of their importance for promoting health benefits perception

Most respondents thought that WPs provided habitat, recreation, air purification, and *water purification* services (Fig. 7a). Habitat and water purification were the two ESs that respondents rated as having the greatest perceived physical and mental health benefits (habitat ranked the first and water purification ranked the second. Air purification was essential for physical health, and recreation was important for mental health (Fig. 7b, c). Education and social relations were least important for promoting perceived health benefits.

##### Wetland Types

Lake was the most popular type among the different wetland types in WPs. Wetland types did not influence the perception of health effects, except for the perception of physical health effects before the Peak and mental health effects during the Peak in regions with the 2^nd^ level RLPHE (Table 3).

**Table 3:** Impact of the factors (wetland parks level) (*: P < 0.05, **: P < 0.01)

		Before Peak		During Peak		After Peak		Difference:	
								After-Before Peak	
	N = 850^a^	Physical	Mental	Physical	Mental	Physical	Mental	Physical	Mental
1st level RLPHE	N = 264								
(highest risk)									
Rivers	36	1.81 ± 1.14	1.81 ± 1.24	0.92 ± 1.73	1.28 ± 1.68	1.75 ± 1.18	1.86 ± 1.27	-0.06 ± 1.12	0.06 ± 0.85
Lakes	182	1.65 ± 1.29	1.74 ± 1.24	0.96 ± 1.57	1.20 ± 1.52	1.82 ± 1.19	1.87 ± 1.18	0.17 ± 1.11	0.13 ± 0.86
Coastal/mangroves	11	1.45 ± 1.04	1.36 ± 1.21	0.73 ± 1.19	0.91 ± 1.30	1.73 ± 1.19	1.73 ± 1.19	0.27 ± 0.65	0.36 ± 0.50
Ponds	5	2.00 ± 0.71	2.20 ± 0.45	0.20 ± 1.30	0.40 ± 1.14	1.80 ± 0.45	2.00 ± 0.00	-0.20 ± 1.10	-0.20 ± 0.45
Rivers + lakes	24	1.96 ± 1.04	2.00 ± 1.02	0.58 ± 1.44	1.17 ± 1.37	2.25 ± 0.74	2.25 ± 0.74	0.29 ± 0.86	0.25 ± 0.79
Mix (≥ 3 types of wetlands)	6	1.67 ± 1.37	2.33 ± 0.82	0.33 ± 2.16	1.00 ± 1.27	2.67 ± 0.82	2.83 ± 0.41	1.00 ± 1.10	0.50 ± 0.55
One-way ANOVA	F	0.474	0.85	0.61	0.393	1.305	1.337	1.226	0.729
	P	0.795	0.515	0.692	0.854	0.262	0.249	0.297	0.602
2nd level RLPHE	N = 228								
(high risk)									
Rivers	28	2.07 ± 1.02	1.93 ± 1.12	1.29 ± 1.54	1.46 ± 1.53	2.25 ± 0.93	2.21 ± 0.92	0.18 ± 0.67	0.29 ± 0.94
Lakes	81	1.70 ± 1.25	1.93 ± 1.12	1.19 ± 1.44	1.62 ± 1.32	1.96 ± 1.12	2.11 ± 1.10	0.26 ± 1.13	0.19 ± 0.95
Coastal/mangroves	32	1.47 ± 1.19	1.53 ± 1.64	1.00 ± 1.67	1.00 ± 1.57	1.66 ± 1.13	1.81 ± 1.12	0.19 ± 0.86	0.28 ± 0.85
Ponds	10	2.00 ± 1.05	2.00 ± 1.25	0.50 ± 1.90	0.60 ± 1.84	2.10 ± 0.99	2.20 ± 0.79	0.10 ± 0.32	0.20 ± 1.23
Swamps	1	-	-	-	-	-	-	-	-
Rivers + lakes	33	1.82 ± 1.19	2.15 ± 1.09	0.85 ± 1.54	1.12 ± 1.34	2.18 ± 1.10	2.18 ± 0.98	0.36 ± 1.08	0.03 ± 0.68
Mix (≥ 3 types of wetlands)	43	2.30 ± 0.94	2.26 ± 1.00	1.37 ± 1.57	1.74 ± 1.33	2.23 ± 1.00	2.21 ± 0.99	-0.07 ± 0.70	-0.05 ± 0.69
One-way ANOVA	F	2.571	1.798	0.884	2.314	1.524	0.715	0.985	0.88
	P	0.028*	0.114	0.493	0.045*	0.183	0.612	0.428	0.496
3rd level RLPHE	N = 358								
(medium risk)									
Rivers	25	1.84 ± 1.21	1.80 ± 1.23	0.84 ± 1.38	1.00 ± 1.56	1.68 ± 1.18	1.68 ± 1.25	-0.16 ± 0.94	-0.12 ± 0.88
Lakes	192	1.80 ± 1.23	1.82 ± 1.25	0.81 ± 1.60	1.03 ± 1.54	1.93 ± 1.14	1.92 ± 1.13	0.13 ± 0.94	0.09 ± 0.87
Coastal/mangroves	3	2.00 ± 1.00	1.67 ± 1.16	2.00 ± 1.00	2.00 ± 1.00	2.33 ± 1.16	2.33 ± 1.16	0.33 ± 1.53	0.67 ± 1.15
Waterfall	2	2.50 ± 0.71	2.50 ± 0.71	0.50 ± 0.71	-1.50 ± 0.71	3.00 ± 0.00	3.00 ± 0.00	0.50 ± 0.71	0.50 ± 0.71
Ponds	9	1.56 ± 1.01	2.00 ± 1.00	1.56 ± 1.13	1.89 ± 1.27	2.11 ± 1.05	2.11 ± 1.05	0.56 ± 0.73	0.11 ± 0.33
Terrace	1	-	-	-	-	-	-	-	-
Mix (≥ 3 types of wetlands)	2	2.00 ± 0.00	1.00 ± 1.41	-0.50 ± 0.71	-0.50 ± 2.12	1.00 ± 1.41	2.00 ± 0.00	-1.00 ± 1.41	1.00 ± 1.41
Rivers + lakes	125	1.73 ± 1.21	1.96 ± 1.15	0.80 ± 1.55	0.97 ± 1.56	1.84 ± 1.08	1.96 ± 1.06	0.11 ± 1.03	0.00 ± 0.90
One-way ANOVA	F	0.255	0.479	0.886	1.924	0.897	0.648	1.164	1.035
	P	0.957	0.824	0.506	0.075	0.497	0.692	0.325	0.402

^a^The names of WPs filled by some respondents failed to be found online due to typos or unclear descriptions. These responses were not included in this part of analysis

#### Individual Level

As shown in Table 4, the 45–54 age group perceived the highest physical and mental health benefits, while the 18–24 age group perceived the lowest physical and mental health benefits (P < 0.05). Men perceived higher health benefits than women (P < 0.05). Education levels and occupational status had no influence on the perceived health effects level before, during and after the Peak. There is a significant difference in the change of perceived health benefits before and after the Peak among various occupational status: compared with before the Peak, the temporarily unemployed and retirees perceived higher mental health benefits than the other two groups after the Peak (P < 0.05).

**Table 4:** Impact of the factors (individual level) (*: P < 0.05, **: P < 0.01)

	N = 967	Before Peak		During Peak		after Peak		Difference:	
								After-before Peak	
		Physical	Mental	Physical	Mental	Physical	Mental	Physical	Mental
Age									
18–24	222	1.54 ± 1.33	1.74 ± 1.22	0.61 ± 1.63	0.92 ± 1.57	1.76 ± 1.19	1.85 ± 1.17	0.22 ± 1.29	0.11 ± 0.96
25–34	360	1.73 ± 1.18	1.80 ± 1.19	0.82 ± 1.46	1.08 ± 1.49	1.85 ± 1.11	1.93 ± 1.10	0.13 ± 0.92	0.13 ± 0.88
35–44	189	1.82 ± 1.18	1.84 ± 1.17	1.10 ± 1.50	1.30 ± 1.41	1.92 ± 1.14	1.92 ± 1.13	0.10 ± 0.83	0.07 ± 0.85
45–54	141	1.82 ± 1.25	1.96 ± 1.22	1.23 ± 1.63	1.41 ± 1.55	2.02 ± 1.19	2.05 ± 1.17	0.20 ± 1.00	0.09 ± 0.68
55–64	46	1.85 ± 1.46	1.78 ± 1.49	0.78 ± 1.76	0.98 ± 1.76	2.13 ± 1.15	2.07 ± 1.16	0.28 ± 1.26	0.28 ± 1.07
≥ 65	9	2.22 ± 1.64	2.44 ± 1.33	1.00 ± 2.35	1.33 ± 2.18	2.00 ± 1.32	2.33 ± 1.12	-0.22 ± 1.48	-0.11 ± 1.36
One-way ANOVA	F	1.799	1.05	3.644	2.487	1.462	0.883	0.783	0.576
	P	0.11	0.387	0.003**	0.030*	0.2	0.492	0.576	0.719
Gender									
Male	416	1.69 ± 1.27	1.81 ± 1.24	1.06 ± 1.51	1.25 ± 1.48	1.90 ± 1.14	1.92 ± 1.12	0.21 ± 1.04	0.11 ± 0.92
Female	551	1.75 ± 1.23	1.83 ± 1.20	0.76 ± 1.61	1.05 ± 1.56	1.87 ± 1.16	1.95 ± 1.15	0.12 ± 1.03	0.12 ± 0.85
Independent samples T test	F	-0.812	-0.183	2.939	2.022	0.396	-0.386	1.42	-0.245
	P	0.417	0.854	0.003**	0.043*	0.692	0.699	0.156	0.807
Highest Education Level									
Junior high school and below	12	1.42 ± 1.31	1.50 ± 1.51	1.00 ± 1.76	0.58 ± 1.83	1.25 ± 1.29	1.58 ± 1.24	-0.17 ± 1.11	0.08 ± 1.38
High school equivalent	50	1.42 ± 1.70	1.62 ± 1.59	0.86 ± 1.86	0.84 ± 1.82	1.82 ± 1.49	1.86 ± 1.43	0.40 ± 1.51	0.24 ± 1.06
Specialized college	87	1.95 ± 1.18	1.84 ± 1.35	0.93 ± 1.72	1.15 ± 1.65	1.98 ± 1.24	2.03 ± 1.18	0.02 ± 0.83	0.20 ± 0.96
Bachelor's	450	1.71 ± 1.25	1.84 ± 1.22	0.86 ± 1.58	1.12 ± 1.53	1.83 ± 1.17	1.89 ± 1.16	0.12 ± 1.04	0.05 ± 0.87
Master's and above	368	1.74 ± 1.18	1.83 ± 1.11	0.90 ± 1.48	1.21 ± 1.44	1.95 ± 1.04	1.99 ± 1.05	0.21 ± 0.98	0.16 ± 0.83
One-way ANOVA	F	1.699	0.599	0.072	1.075	1.639	0.907	1.757	1.287
	P	0.148	0.664	0.99	0.368	0.162	0.459	0.135	0.273
Occupation Status									
Student	235	1.60 ± 1.26	1.84 ± 1.15	0.69 ± 1.61	1.04 ± 1.61	1.80 ± 1.14	1.92 ± 1.14	0.20 ± 1.20	0.08 ± 0.92
Employee	647	1.77 ± 1.22	1.83 ± 1.20	0.95 ± 1.53	1.17 ± 1.49	1.89 ± 1.15	1.92 ± 1.14	0.12 ± 0.94	0.09 ± 0.82
No occupation	41	1.51 ± 1.36	1.71 ± 1.47	1.17 ± 1.50	1.41 ± 1.32	2.02 ± 1.17	2.10 ± 1.00	0.51 ± 1.29	0.39 ± 1.12
Retiree	44	1.89 ± 1.37	1.68 ± 1.55	0.64 ± 1.91	0.84 ± 1.89	2.05 ± 1.22	2.07 ± 1.23	0.16 ± 1.12	0.39 ± 1.19
One-way ANOVA	F	1.832	0.353	2.408	1.372	0.915	0.515	2.096	3.041
	P	0.14	0.787	0.066	0.244	0.433	0.672	0.099	0.028*
Professional									
Architecture & built environment	432	1.78 ± 1.15	1.88 ± 1.17	0.82 ± 1.49	1.09 ± 1.44	1.91 ± 1.08	1.97 ± 1.07	0.14 ± 0.94	0.10 ± 0.93
Art & design	81	1.35 ± 1.49	1.56 ± 1.31	0.64 ± 1.60	0.98 ± 1.59	1.74 ± 1.24	1.78 ± 1.20	0.40 ± 1.51	0.22 ± 0.96
Hydrology	16	1.50 ± 1.27	1.69 ± 1.25	1.00 ± 1.37	1.31 ± 1.14	1.63 ± 1.46	1.69 ± 1.49	0.13 ± 0.72	0.00 ± 0.63
Psychology	9	2.00 ± 1.23	1.89 ± 1.45	0.89 ± 2.26	1.44 ± 1.42	1.89 ± 1.27	2.11 ± 1.27	-0.11 ± 0.78	0.22 ± 0.44
Medicine, nursing	47	1.70 ± 1.25	1.43 ± 1.33	0.66 ± 1.49	0.81 ± 1.56	1.51 ± 1.32	1.53 ± 1.28	-0.19 ± 1.04	0.11 ± 0.96
Agriculture/forestry	45	1.44 ± 1.16	1.62 ± 1.19	1.07 ± 1.25	1.22 ± 1.43	1.67 ± 1.17	1.80 ± 1.08	0.22 ± 0.74	0.18 ± 0.81
Environmental science	21	2.24 ± 0.83	2.33 ± 0.86	1.24 ± 1.76	1.29 ± 1.77	2.43 ± 0.68	2.38 ± 0.74	0.19 ± 0.51	0.05 ± 0.38
Social science	51	1.53 ± 1.35	1.61 ± 1.33	0.76 ± 1.77	0.96 ± 1.71	1.75 ± 1.16	1.90 ± 1.15	0.22 ± 1.24	0.29 ± 1.17
Economy & finance	45	2.02 ± 1.26	2.18 ± 1.10	0.91 ± 1.78	0.86 ± 1.86	2.13 ± 0.95	2.12 ± 0.96	0.11 ± 1.23	-0.02 ± 0.80
Others	220	1.78 ± 1.32	1.89 ± 1.23	1.11 ± 1.66	1.37 ± 1.56	1.95 ± 1.23	1.98 ± 1.22	0.18 ± 1.02	0.09 ± 0.75
One-way ANOVA	F	2148	2.342	1.077	1.227	1.903	1.652	1.219	0.609
	P	0.023*	0.013*	0.377	0.274	0.048	0.096	0.279	0.79
Health experts	56	1.75 ± 1.24	1.50 ± 1.35	0.70 ± 1.62	0.91 ± 1.54	1.57 ± 1.31	1.63 ± 1.29	-0.18 ± 0.99	0.13 ± 0.90
(Medical and nursing, psychology)									
Others	911	1.72 ± 1.25	1.84 ± 1.21	0.90 ± 1.57	0.15 ± 1.53	1.90 ± 1.14	1.94 ± 1.14	0.18 ± 1.03	0.11 ± 0.88
Independent samples T test	F	0.155	-2.052	-0.927	-1.124	-0.081	-2.122	-2.51	0.098
	P	0.877	0.040*	0.354	0.261	0.038*	0.034*	0.012*	0.922

Groups with various professional backgrounds had significant differences in the perceived health benefits before the epidemic (P < 0.05); groups with environmental science backgrounds had the highest level of perceived health benefits. In addition, health experts (i.e., persons with medical, nursing, and psychology backgrounds) had significantly lower perceptions of mental health benefits before the epidemic and physical and mental health benefits after the Peak than other professional groups (P < 0.05). Meanwhile, health experts believed that the health benefits after the Peak were slightly lower than those before the epidemic, which was opposite to other groups of people.

The self-reported physical health status before the epidemic was positively correlated with the perceived physical health benefits before the Peak (Pearson correlation = 0.06, P < 0.05, see Table 5). The perceived physical or mental health benefits during and after the Peak were not statistically correlated with the self-reported physical or mental health status on the survey day. Also, the change of perceived health benefits was not statistically correlated with the change of self-reported health status.

**Table 5:** Correlation between self-reported health status and perceived health benefit (N = 967, *: P < 0.05)

Physical Health			Self-reported physical health status		
			Before Peak	Current	Difference: current-before
perceived health benefit	Before Peak	Pearson Correlation	0.069*	-	-
		Sig.(2-tailed)	0.031	-	-
	During Peak	Pearson Correlation	-	0.004	-
		Sig.(2-tailed)	-	0.903	-
	After Peak	Pearson Correlation	-	0.03	-
		Sig.(2-tailed)	-	0.356	-
	Difference: after-before	Pearson Correlation	-	-	-0.004
		Sig.(2-tailed)	-	-	0.91
Mental Health			Self-reported mental health status		
			Before Peak	Current	Difference: current-before
Perceived health benefit	Before Peak	Pearson Correlation	0.062	-	-
		Sig.(2-tailed)	0.055	-	-
	During Peak	Pearson Correlation	-	-0.004	-
		Sig.(2-tailed)	-	0.893	-
	After Peak	Pearson Correlation	-	0.006	-
		Sig.(2-tailed)	-	0.85	-
	Difference: after-before	Pearson Correlation	-	-	-0.032
		Sig.(2-tailed)	-	-	0.314

## Discussion

In general, the public perceives wetlands to be beneficial for physical and mental health, which is consistent with the conclusion of previous studies that urban green space and blue-green space are beneficial to people's physical and mental health (see "Health Effects of Wetland Parks and Ecosystem Services" section). A possible reason for the lowest perceived health benefits during the Peak could be the higher risk of infection. Limited access to WPs during the Peak could also contribute to the low perception of health benefits. The increase in perceived health benefits after the Peak shows that inaccessibility to WPs for a period of time may improve perceived health benefits from WPs.

On the city level, results show that a moderate epidemic risk stimulates perception of physical and mental health benefits from WPs. Further investigation regarding health benefits and harm perception associated with epidemic risks is needed to draw more precise recommendations for further improvement of WPs from the perspective of public health.

On the community level, unexpectedly, the perceived level of physical and mental health benefits after the Peak is negatively associated with the lockdown degree of the community, suggesting that quarantine did not lead to an increase in health-related motivation for visiting WPs.

In terms of WPs level, habitat services were considered to be the most important ecosystem services that promote the perceived health benefits. The possible reasons are: (1) self-reported happiness is positively correlated with the perceived species richness of birds, butterflies, and plants (Dallimer et al. 2012); (2) the biologically diverse natural environment can improve health by exposure to a pleasant environment or encouraging health promotion behaviours (Lovell et al. 2014); (3) there is a strong positive correlation between vegetation cover and personal well-being. The relationship between human well-being and nature is weakly correlated with changes in species richness, bird abundance, and plant density (Luck et al. 2011). However, habitat services were regarded as indirect health-related ES that affect human health through another service, and the mechanism of their effect on health is still unclear. The importance of habitat, air purification and recreation services align with the motivation for visiting WPs (e.g., being close to nature and wildlife habitat, enjoying fresh air and going out for exercises).

On the individual level, this study has found that men perceive higher health benefits than women when visiting urban blue-green spaces during the Peak. There is no significant gender difference before and after the epidemic. This is different from the result of a previous study based on two of Canada's blue-green spaces that women usually perceive higher health well-being than men from visiting nature reserves (Lemieux et al. 2012). The phenomenon that housewives and the elderly are more dependent on the local environment and therefore are more susceptible to the local environment (de Vries et al. 2003) could be a possible explanation to our result that the temporarily unemployed (e.g. housewives) and retirees (e.g. the elderly) perceived higher mental health benefits than the other two groups after the Peak. In addition, health experts' perception of mental health benefits before the epidemic and that of physical and mental health after the Peak were significantly lower than other professional groups, which indicate that lay people may have overestimated or health experts may have underestimated the health benefits of visiting WPs. Besides, health experts believe that the health benefits after the peak of the epidemic are slightly lower than before the outbreak, while other people have the opposite view. This may be because health experts believe that travel after the peak of the epidemic poses a higher risk.

This study is based on a large number of subjective responses regarding the perceived health effects of WPs. It does not objectively measure the health effects of WPs. Ecosystem disservices could negatively affect the perception of health benefits. For conducting the questionnaire, it was the assumption that there is little risk of infection by COVID-19 when visiting wetland parks after the Peak, which naturally excludes the effect of some infectious disease-related ecosystem disservices on health perception. Moreover, factors such as the quality, area, and naturalness of the WPs may affect health (Ekkel and de Vries 2017), and perceived health benefits. This study is a general analysis based on national sampling. It does not provide a detailed analysis of specific WPs, including their quality, area, and naturalness. To control the number of questions and response time, this study did not use more detailed assessment scales (e.g. EQ-5D (Leidl 2009), General Health Questionnaire (White et al. 2013)) to assess health status. This could have an influence on the respondents' self-reported health status. Most participants had a high level of education, suggesting that they understand the contents of the questionnaire well. Due to the restrictions in face-to-face survey and the suspended express delivery in high-epidemic-risk regions during the pandemic, face-to face and mail surveys were not applicable. Besides, telephone surveys were usually rejected as fraudulent calls. Thus, this study relies on the online survey which was the most feasible method for collecting as many data from all over the country as possible within a very short period of time, potentially making it difficult for the elderly and non-internet users to get involved.

## Conclusion

This research gives an overview of the perceived health effects of WPs in the context of COVID-19 in China to contribute to existing knowledge of health benefits of urban blue and green spaces and the link between ESs and human health. This study confirms that most people can perceive the health benefits of WPs. A slightly higher level of perceived health benefits after the Peak than before indicates that limited access to WPs increases perceived health benefits. Whilst very high epidemic risks might be said to have a negative impact on perceived physical health benefits, and lower risks may not be conducive to perceived mental health benefits, the moderate epidemic risk seems to be associated with greater physical and mental health benefits for visiting WPs during the Peak. Also, quarantine did not lead to an increase in health-related motivation for visiting WPs. Interestingly, health experts perceived lower health benefits than laypeople. At the same time, habitat services, which were regarded as indirect health-related ES, were perceived as the most crucial ES for promoting the perceived health benefits in WPs. Overall, a better understanding of the perception of health benefits of WPs can help to provide empirical evidence about ecosystem services as delivered by WPs, or green and blue space in general, in the context of COVID-19 and also regarding potential future pandemics.

## Data Availability

The datasets generated and/or analysed during the current study are available in the Figshare repository, https://figshare.com/s/3fb0f4d39f6459c4bba7

## Footnotes

1 The spokesperson of the National Health Commission of the People’s Republic of China declared that “China has passed the climax of the novel coronavirus outbreak, with the number of new infections continuing to decline” on March 12nd,2020.

2 We set the questionnaire distribution on the Tencent Questionnaire Platform to send automatic reminders for every 50 new responses, and manually checked and eliminated invalid questionnaires (such as questionnaires answered by children) simultaneously with the collection of questionnaires. We closed the questionnaire immediately after receiving 1,400 valid responses.

3 When the survey was conducted, the epidemic was expected to end by April 2020. However, this epidemic subsequently developed into a global pandemic and has not yet ended. Therefore, in the questionnaire, "during the epidemic" refers to the Peak, and "after the epidemic" and "when the epidemic is over" mean "after the Peak". Because the survey period was when this new coronavirus was first identified after it had spread throughout China, cities across the country have taken the same epidemic prevention measures at almost the same time.

## Abbreviations

WPs: Wetland parks
ESs: Ecosystem services
Peak: The peak of the COVID-19 pandemic
